# Single-cell antibody nanowells: a novel technology in detecting anti-SSA/Ro60- and anti-SSB/La autoantibody-producing cells in peripheral blood of rheumatic disease patients

**DOI:** 10.1186/s13075-016-1010-5

**Published:** 2016-05-17

**Authors:** Lida Esfandiary, Nirupama Gupta, Alexandria Voigt, Arun Wanchoo, Edward K. L. Chan, Sukesh Sukumaran, Cuong Q. Nguyen

**Affiliations:** Department of Infectious Diseases and Pathology, College of Veterinary Medicine, University of Florida, PO Box 110880, Gainesville, FL 32611-0880 USA; Department of Pediatric Nephrology, University of Florida, Gainesville, FL USA; Department of Oral Biology, University of Florida, Gainesville, FL USA; Rheumatology Section, Department of Pediatrics, University of Arkansas for Medical Sciences, Arkansas Children’s Hospital, Little Rock, AR USA; Center of Orphaned Autoimmune Diseases, University of Florida, Gainesville, FL USA

**Keywords:** Sjögren’s syndrome, Systemic lupus erythematosus, Antinuclear autoantibody, Anti-SSA/Ro60, Anti-SSB/La, Single-cell analysis

## Abstract

**Background:**

Anti-SSA/Ro60 and anti-SSB/La are essential serological biomarkers for rheumatic diseases, specifically Sjögren’s syndrome (SS) and systemic lupus erythematosus (SLE). Currently, laboratory detection technology and platforms are designed with an emphasis on high-throughput methodology; therefore, the relationship of sensitivity with specificity remains a significant area for improvement. In this study, we used single-cell antibody nanowells (SCAN) technology to directly profile individual B cells producing antibodies against specific autoantigens such as SSA/Ro60 and SSB/La.

**Methods:**

Peripheral blood mononuclear cells were isolated using Ficoll gradient. Fluorescently labeled cells were added to fabricated nanowells and imaged using a high-speed epifluorescence microscope. The microengraving process was conducted using printed slides coated with immunoglobulins. Printed slides were hybridized with fluorescence-conjugated immunoglobulin G (IgG), SSA/Ro60, and SSB/La antigens. Microarray spots were analyzed for nanowells with single live B cells that produced antigen-specific autoantibodies.

**Results:**

Our results indicate that SCAN can simultaneously detect high frequencies of anti-SSA/Ro60 and anti-SSB/La with a specific IgG isotype in peripheral blood mononuclear cells of patients, as well as measure their individual secretion levels. The data showed that patients with SS and SLE exhibited higher frequency and greater concentration of anti-SSA/Ro60- and anti-SSB/La-producing B cells in the IgG isotype. Furthermore, individual B cells of patients produced higher levels of IgG-specific anti-SSA/Ro60 autoantibody, but not IgG-specific anti-SSB/La autoantibody, compared with healthy control subjects.

**Conclusions:**

These results support the application of SCAN as a robust multiparametric analytical bioassay that can directly measure secretion of autoantibody and accurately report antigen-specific, autoantibody-producing cells.

## Background

Sjögren’s syndrome (SS) and systemic lupus erythematosus (SLE) patients develop specific autoantibodies against nuclear antigens, intracellular components, membrane proteins, and secreted products of exocrine tissues [1–5]. Between 40 % and 70 % of SS patients’ sera contains autoantibodies that are reactive to SSA/Ro60 and/or SSB/La antigens [6]. Patients with SLE develop similar autoantibodies with lower frequencies (anti-SSA/Ro60 30–40 %, anti-SSB/La 10–15 %) [5]. Timely and accurate measurement of these signature biomarkers is essential for disease diagnosis, prognosis, extraglandular disease classification, and the assessment of treatment outcomes [7]. A seminal study by Jonsson et al. [8] clearly demonstrated that among 625,000 patients studied, those who were presymptomatic developed autoantibodies many years before the clinical onset of the disease. A recent study by Theander et al. [9] indicated a timeline of 18–20 years before diagnosis of primary SS (pSS) in which autoantibodies are present. In a recent study of the National Health and Nutrition Examination Survey cohort, anti-SS-B/La or anti-SSA/Ro60 was confirmed to be uncommon in this representative U.S. population [10]. While there is still speculation whether these autoantibodies always develop before disease, new, innovative techniques with high sensitivity and specificity will allow researchers to study this process in more detail at an individual cell level.

Autoantibody screening by routine laboratory techniques uses mainly serum as the source. Immunostaining of the salivary glands in patients with pSS could identify the presence of anti-SSA/Ro60 and anti-SSB/La autoantibody-producing cells [11]. Recent advances in recombinant monoclonal antibody technology have significantly expanded understanding of the broad autoantibody profile detected in the glands [12]. A number of methods are used for the detection of anti-SSA/Ro60 and anti-SSB/La antibodies, such as double-immunodiffusion, counterimmunoelectrophoresis, Western blotting, immunoprecipitation, and enzyme-linked immunosorbent assay (ELISA) [13]. Recent advances in addressable laser bead immunoassays that use color-coded microspheres conjugated with an antigen of interest have increased the feasibility of the high-throughput analyses for multiple antigens simultaneously [14]. These assays have provided valuable patient data that are beneficial in diagnosis and treatment. However, the major drawback for some of these techniques, such as the laser bead assay, is the high sensitivity, which could result in false-positive results [15, 16]. Commonly used autoantibody assays use sera to examine the antibody profile instead of directly examining the B-cell source of these secreted antibodies.

In this study, we used single-cell antibody nanowells technology to evaluate the specific autoantibodies produced by individual live B cells in a high-throughput, highly specific process. SCAN technology involves a soft microengraving technique that uses a dense array of nanowells (50 × 50-μm wells holding a volume of 0.1–1 nl each) fabricated of polydimethylsiloxane (PDMS) to isolate individual cells for printing of corresponding molecules secreted by each cell [17]. The results of this study indicate that SCAN technology is able to detect single live B cells that produce higher levels of anti-SSA/Ro60 and anti-SSB/La in pSS and SLE patients. In addition, the results demonstrate that SCAN technology is able to enumerate the frequency and quantify the concentration of anti-SSA/Ro60 and anti-SSB/La from individual B cells isolated from subjects’ peripheral blood mononuclear cells (PBMCs). As a research tool, SCAN technology is capable of interrogating individual, unique, antibody-secreting B cells. Further studies are needed to validate it as a diagnostic tool for patient classification.

## Methods

### Human subjects

Participants underwent extensive serologic evaluations as standard of care. The serological tests were analyzed using ELISA for anti-SSA/Ro (QUANTA Lite® SS-A, catalog number 708570; Inova Diagnostics, San Diego, CA, USA); anti-SSB/La QUANTA Lite® SS-B, catalog number 708575; Inova Diagnostics); rheumatoid factor (Inova Diagnostics); and anti-Smad, anti-ribonucleoprotein, anticentromere, anti-Scl-70, and double-stranded DNA (Bio-Rad Laboratories, Hercules, CA, USA). Indirect immunofluorescence was used for Hep2 cells (antinuclear antibodies [ANAs]; Bio-Rad Laboratories), chemiluminescence immunoassay for anticardiolipin (Inova Diagnostics), and immunoturbidimetry for complement components C3 and C4. PBMCs and blood (*n* = 9 healthy control subjects aged 17–65 years, with 8 females and 1 male; *n* = 13 pSS/SLE patients with clinical profiles presented in Table 1) were either generously given by the Sjögren’s International Collaborative Clinical Alliance (SICCA) or obtained at the University of Florida Department of Pediatric Rheumatology. PBMCs from the SICCA registry were frozen samples and stored in cryoprotective media containing 90 % fetal calf serum and 10 % dimethyl sulfoxide. To maintain consistency, blood samples obtained at the University of Florida were first processed by Ficoll-Paque PLUS as instructed by the manufacturer (GE Healthcare Life Sciences, Piscataway, NJ, USA). Isolated PBMCs were frozen in cryoprotective media. Before being screened for autoantibody production, PBMCs were thawed and rested for 1 h in complete media (RPMI 1640 medium, 10 % FBS, 2 mM l-glutamine, 0.05 mM β-mercaptoethanol, 37 °C, 5 % CO_2_). Cell viability was determined to be 70–85 % using 0.4 % Trypan Blue dye (Bio-Rad Laboratories) and read with an automated cell counter (TC20; Bio-Rad Laboratories). All procedures were reviewed and approved by the University of Florida Health Institutional Review Board (IRB201400079). Written informed consent was obtained from study participants before enrollment in the study.

**Table 1:** Patients’ clinical profiles

Patient	Age (years)	Sex	RF (IU/ml)	Disease	Focus score	ANA	Anti-Ro	Anti-La
2.4	16	M	N/A	SLE	N/A	+	−	−
2.24	15	F	<10	SLE	N/A	−	−	−
3.9	8	F	16.5	SLE	N/A	1/80	+	−
3	16	F	N/A	SLE	N/A	+	+	+
4.1	11	F	N/A	SLE	N/A	+	+	+
14	16	F	12	SLE	N/A	+	−	+
15	17	F	<10	SLE	N/A	+	−	−
24	15	F	<10	SLE	N/A	−	−	−
25	16	F	<10	SLE	N/A	+	−	−
26	16	F	86	SLE	N/A	+	+	+
27	15	F	<10	SLE, sSS	N/A	+	+	+
1	48	F	+	pSS	2.7	1/320	+	+
2	56	F	−	pSS	2.3	1/320	+	+

*ANA* antinuclear autoantibodies, *pSS* primary Sjögren’s syndrome, *RF* rheumatoid factor, *SLE* systemic lupus erythematosus, *RF* rheumatoid factor, *sSS* secondary Sjögren’s syndrome, *N/A* not applicable, *IU/ml* international units per milliliter

### Preparation and imaging of loaded cells in arrays of nanowells

Nanowells were fabricated using SYLGARD 184 silicone elastomer base (PDMS; Dow Corning, Midland, MI, USA) and a curing agent as described previously [17]. A suspension of 2 × 10^5^ cells in 100 μl of complete culture media was stained with CellTrace Calcein Violet, AM, for live cells and anti-CD19-Alexa Fluor (AF) 488 for 30 minutes on ice (Life Technologies, Carlsbad, CA, USA). Stained cells were washed in PBS and resuspended in 300 μl of culture media. The suspension of cells was loaded into an array of 50-μm nanowells. The cells were allowed to settle via gravity for 5 minutes. Excessive cells were rinsed off with media, and a LifterSlip coverslip (Fisher Scientific, Pittsburgh, PA, USA) was placed on top to prevent evaporation from the nanowells. The arrays were imaged using an automated epifluorescence microscope (Nikon Eclipse Ti; Nikon Instruments, Melville, NY, USA) equipped with a motorized stage, phase contrast, and 405-nm and 488-nm wavelength filter sets using Nikon NIS Elements Advanced Research image capture software.

### Microengraving

The array of nanowells was submerged in media to be rewetted after imaging. The array was then gently placed in the chamber base of a hybridization chamber (Agilent Technologies, Santa Clara, CA, USA), and excess liquid was aspirated off using a glass pipette. The face of each dry glass slide treated with poly-l-lysine as described previously [17] was coated with polyclonal donkey antihuman immunoglobulin G (IgG) (25 μg/ml; Jackson ImmunoResearch, West Grove, PA, USA) was placed on top of the array, which was placed inside the chamber base. The assembly was secured by a finger-tightened screw and incubated at 37 °C for 1 h. After incubation, the glass slides were carefully removed from the array and immediately placed in 1× PBS. The glass slides were processed using the HS 400 Pro Hybridization system (Tecan, Männedorf, Switzerland) with the following protocol: 15-minute hybridization with 3 % nonfat milk in PBS with 0.5 % Tween 20, washed twice for 1 minute each time, and incubation for 45 minutes with a 1:1000 dilution of goat antihuman IgG-AF647 (Jackson ImmunoResearch), AF488-labeled SSA/Ro60, and AF550-labeled SSB/La using DyLight antibody labeling kits per the manufacturer’s instructions (Thermo Scientific, Rockford, IL, USA). Ro60/SS-A and La/SS-B were purchased from a commercial source (DIARECT, Freiburg, Germany). The slides were vacuum-dried and scanned using a GenePix 4400 microarray scanner (Molecular Devices, Sunnyvale, CA, USA) with specific gain and power to maintain consistency between fluorescence channels as well as among subsequent slides.

### Data analysis

Microarray micrographs and microscopic images were processed to identify nanowells containing single cells with corresponding secretion of IgG antibody against Ro60/SS-A and La/SS-B proteins. In brief, GenePix Pro-7.0 software (Molecular Devices) was used to locate positive features on the scanned images of printed microarrays using a custom GenePix Array List designated with feature indicators. Once all the features were found, each position in the array was analyzed to extract the mean fluorescence intensity (MFI) for each channel corresponding to immunoglobulin. The data were extracted on the basis of specific criteria for each channel. This included setting the percentage of saturation to any value less than 2, the coefficient of variation to a maximum of 100 to indicate signal uniformity, the signal-to-noise ratio to greater than or equal to 1, and the SD above the background (% > B + 2 SD) at a minimum of 50. These criteria ensured that affirmative and uniform signals above the background noise were selected and reduced the chances for false-positive results. Analysis of the images of the cells recorded by automated epifluoresence microscopy were inspected by using a custom software program to determine the number of cells present in each well and the MFI in each of the fluorescent channels. These data were matched with the corresponding antibodies detected by microengraving according to the unique location identification of each nanowell. This combined dataset was then filtered for analysis to include only wells occupied with single live cells.

### Determine the concentration of anti-SSA/Ro60 and anti-SSB/La

A standard curve for the antibodies produced by each cell was constructed by applying a series of concentrations (e.g., 1 nM to 10 μM) of the corresponding fluorochrome-conjugated antihuman IgG (antihuman IgG-AF488 for SSA/Ro60 and antihuman IgG-AF550 for SSB/La) to the set of replicate microarrays and then measuring the fluorescence intensities of captured anti-SSA/Ro60 and anti-SSB/La as a function of concentration [18, 19]. Based on the standard curve, the concentrations of anti-SSA/Ro60 and anti-SSB/La were calculated using MFI of individual signals on the microarray from each CD19^+^ B cell in nanowells. Based on the unknown concentrations and the MFI values, the linear regression analysis generated for anti-SSA/Ro60 was *y* = 0.0008*x* + 0.0122, *R*^2^ = 0.97609, and for anti-SSB/La it was *y* = 205,706*x* + 124.22, *R*^2^ = 0.99995.

### Statistical analysis

Statistical evaluations were performed by using the Mann-Whitney *U* test generated using InStat software (GraphPad Software, La Jolla, CA, USA). A one-tailed *p* value less than 0.05 was considered significant.

## Results

### Determining anti-SSA/Ro60- and anti-SSB/La-producing B cells using SCAN technology

**Fig. 1:**
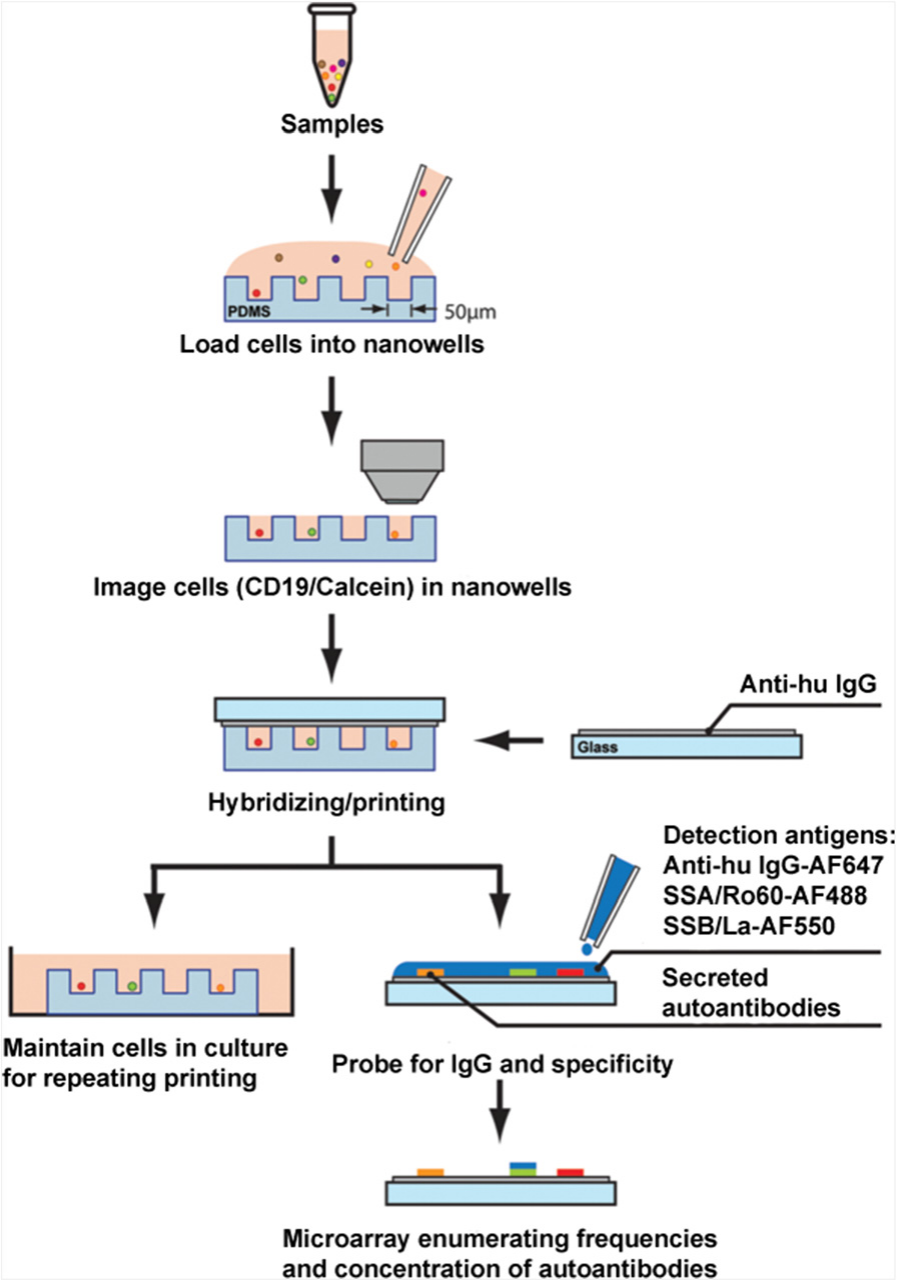
Single-cell antibody nanowell process. Arrays of nanowells with dimensions of 50 μm × 50 μm × 50 μm were used for microengraving. Peripheral blood mononuclear cells were loaded into the nanowells. Cells in the nanowells were imaged using an automated epifluorescence microscope. Micrograving was performed by hybridizing nanowells with capture slides containing antihuman immunoglobulins for 1 h at 37 °C with 5 % CO_2_. After incubation, nanowells containing intact live cells and capture slides were separated. A mixture of goat antihuman immunoglobulin G (IgG)-Alexa Fluor 647 (AF647) and fluorochrome-conjugated SSA/Ro60-AF488 and SSB/La-AF550 were added to the capture slides. Micrographs of microarrays were generating by scanning using a GenePix Autoloader 4200AL microarray scanner. The schematic has been modified from a previous study [17]

**Fig. 2:**
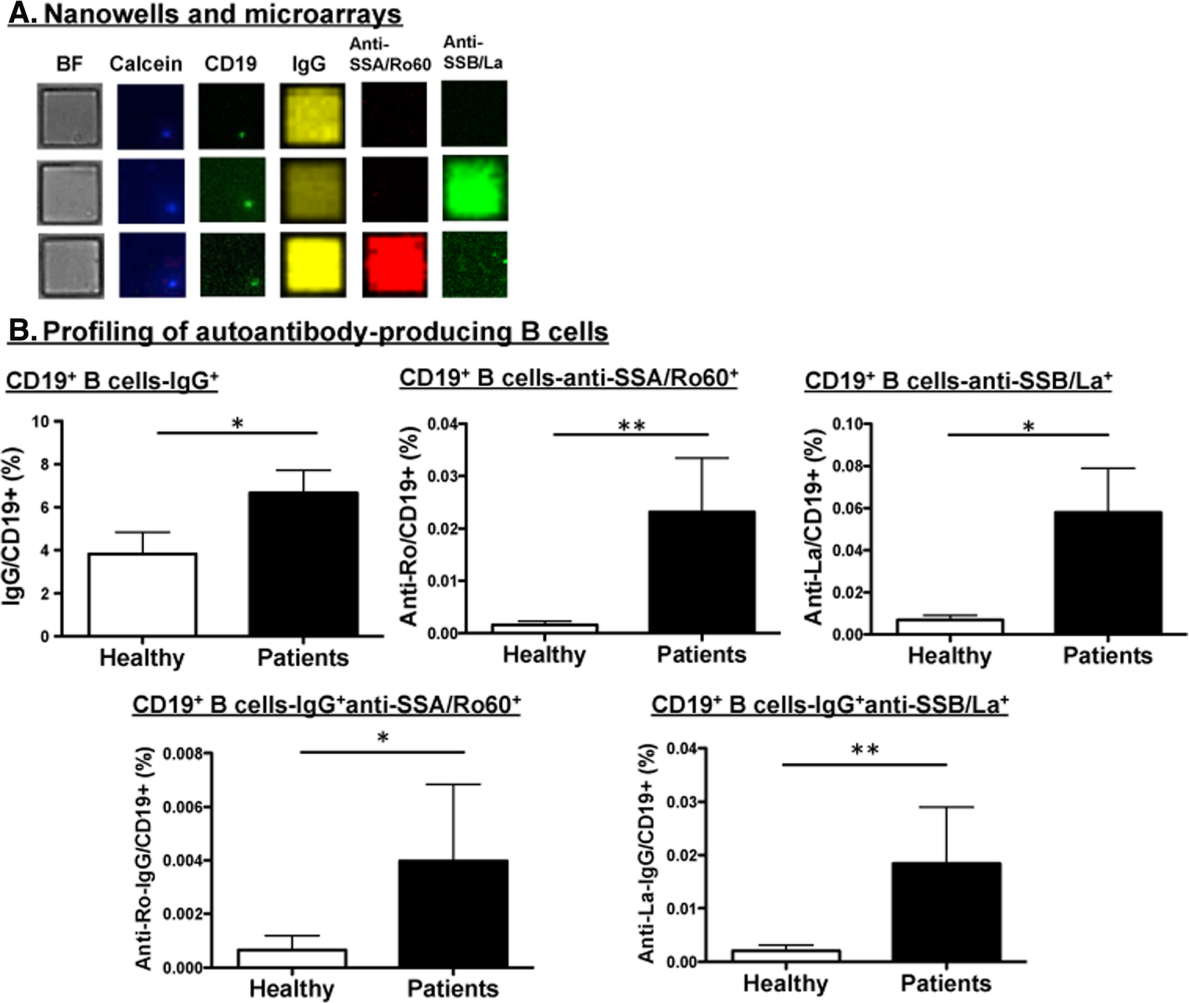
Profiling anti-SSA/Ro60- and anti-SSB/La-producing B cells using single-cell antibody nanowell technology. **a** Representative micrographs of cells (bright field [BF]) in nanowells labeled with calcein (live cells) and CD19-Alexa Fluor 488 (AF488). Micrographs of matching microarray by microengraving showing detection signals for immunoglobulin G (IgG)-AF647, anti-SSA/Ro60-AF488, and anti-SSB/La-AF550. **b** Profiling of autoantibody-producing B cells in nanowells (*n* = 9 healthy control subjects, *n* = 13 pSS/SLE patients). Data extracted by image processing with GenePix software were used to identify the appropriate signals. The data were correlated with the nanowell image data in which nanowells contained a single cell positive for both calcein and CD19. The percentage of CD19^+^ B cells producing IgG, anti-SSA/Ro60, anti-SSB/La, anti-SSA/Ro60 in IgG isotype, and anti-SSB/La in IgG isotype was determined by using the count of positive signals from wells with single cells and the total number of wells with single cells. *p < 0.05, **p < 0.01 by unpaired *t* test

To demonstrate the feasibility of SCAN methodology, PBMCs from pSS/SLE patients and healthy control subjects were isolated. As presented in Fig. 1, the fluorescently labeled cells were dispersed into the nanowells and imaged to precisely locate specific nanowells that contained individual live B cells. Capture slides containing human anti-IgG were used to hybridize the arrays containing cells. To identify specific captured Ig, detection reagents, including antihuman IgG-AF647, SSA/Ro60-AF488, and SSB/La-AF550, were used to determine the antigen specificity of B cells isolated from patients and healthy control subjects. As demonstrated in Fig. 2a, with SCAN technology we were able to identify an individual live CD19^+^ B cell in each nanowell, but, more importantly, we were able to analyze the IgG-specific autoantibodies produced by these individual CD19^+^ B cells, as represented by IgG, anti-SSA/Ro60, and anti-SSB/La. On the basis of the microarrays of the secreted autoantibodies, nanowells containing a single CD19^+^ B cell were identified to be either negative for anti-SSA/Ro60 and anti-SSB/La or positive for anti-SSA/Ro60 or anti-SSB/La, but not both. This observation points to the astute specificity of SCAN technology in detecting anti-SSA/Ro60 and anti-SSB/La autoantibodies.

The application of SCAN technology to PBMCs of pSS/SLE patients or healthy control subjects shows a quantitative difference between binding of SSA/Ro60 and SSB/La antigens. As presented in Fig. 2b, when normalized against nanowells that contained single live cells, patients showed a higher number of CD19^+^ B cells producing IgG in comparison to healthy control subjects. In addition, CD19^+^ B cells of patients produced a higher frequency of anti-SSA/Ro60 and anti-SSB/La autoantibodies than those of healthy control subjects. Combining the two datasets indicated that patients’ CD19^+^ B cells secreted significantly elevated levels of anti-SSA/Ro60 and anti-SSB/La autoantibodies with IgG isotype in comparison to control subjects. The striking aspect of these results is the detection of anti-SSA/Ro60 and anti-SSB/La autoantibodies in healthy control subjects, which were determined to be negative for autoantibodies by ELISA. These results demonstrate that SCAN is a sensitive and multiparametric technology with high specificity that can be used to profile the isotype and autoantibody specificity of individual B cells in patients with pSS and patients with SLE.

### Quantifying the concentration of anti-SSA/Ro60 and anti-SSB/La produced by individual B cells

**Fig. 3:**
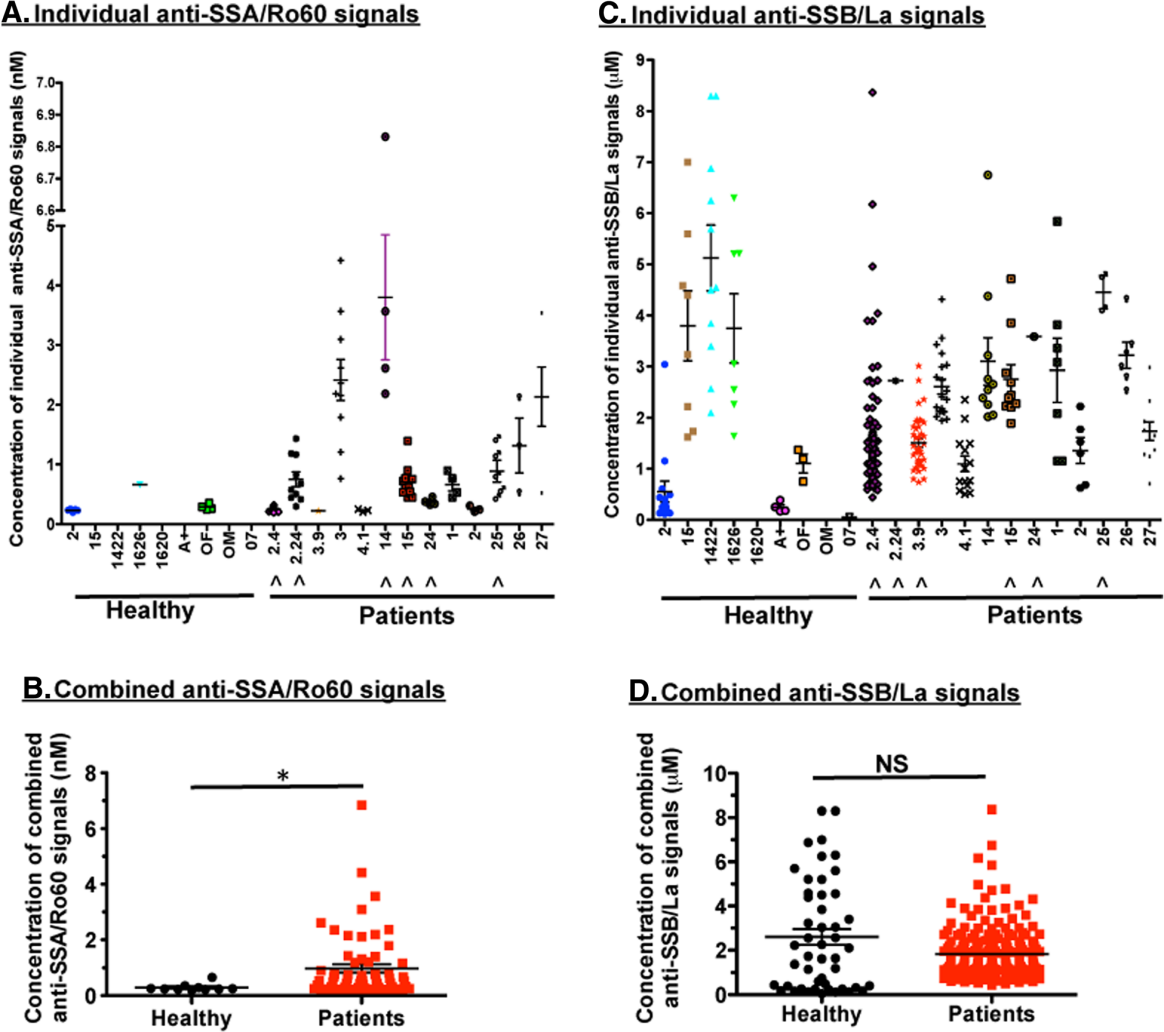
Quantifying the concentration of anti-SSA/Ro60 and anti-SSB/La produced by individual B cells. A standard curve for the antibodies produced by each cell was constructed by applying a series of concentrations of corresponding fluorochrome-conjugated antihuman immunoglobulin G (IgG) (e.g., 1 nM to 10 μM) to the set of replicate microarrays and then measuring the mean fluorescence intensity of captured anti-SSA/Ro60 and anti-SSB/La as a function of concentration using data processing of micrographs of microarrays. **a** Concentration of each signal spot from nanowells with single live CD19^+^ B cell producing IgG-specific anti-SSA/Ro60. ^^^Seronegative patients for anti-SSA/Ro60 autoantibody. **b** Combined concentrations of individual positive signal spots (live CD19^+^ B cells secreting IgG-specific anti-SSA/Ro60 autoantibody) from patients and healthy control subjects. **c** Concentration of each signal spot from nanowells with single live CD19^+^ B cell producing IgG-specific anti-SSB/La. ^^^Seronegative patients for anti-SSB/La autoantibody. **d** Combined concentrations of individual positive signal spots (live CD19^+^ B cells secreting IgG-specific anti-SSB/La autoantibody) from patients and healthy control subjects. X-axis in (**a**) and (**c**) denotes healthy and patient coded personal identification. *p < 0.05. *NS* not significant by unpaired *t* test

The standard curves were generated to calculate the concentration of each anti-SSA/Ro60 and anti-SSB/La signal. As presented in Fig. 3a, individual CD19^+^ B cells of all patients produced various concentrations of anti-SSA/Ro60 based on individual microarray spots. Interestingly, three of nine (patient number 2, 1626, and OF) of the healthy subjects were shown to produce anti-SSA/Ro60 autoantibody, but at significantly lower concentrations (calculated by spot signal intensity) than the patients (Fig. 3b). Six of thirteen of the patients (patient numbers 2.4, 2.24, 14, 15, 24, and 25 in Table 1) were positive for anti-SSA/Ro60 by SCAN technology, even though they tested negative for anti-SSA/Ro60 by ELISA. Similarly, individual B cells of pSS/SLE patients secreted different amounts of anti-SSB/La autoantibody (Fig. 3c). Some of the anti-SSB/La-negative patients (6 [46 %] of 13) (Table 1) produced large amounts of anti-SSB/La autoantibody determined by using SCAN technology. Analysis of the healthy control subjects revealed that six of nine healthy subjects secreted significant levels of anti-SSB/La autoantibody with overall concentration equivalent to that of the patients (Fig. 3d). The results indicate that SCAN is capable of precisely quantifying the amount of anti-SSA/Ro60 and anti-SSB/La autoantibodies produced by individual B cells. In addition, the data indicate that patients produced higher concentrations of anti-SSA/Ro60 autoantibodies in comparison to healthy control subjects. Last, the secreted levels of anti-SSA/Ro60 autoantibodies by individual B cells appeared to be highly elevated in pSS/SLE patients; however, there was no change in the amount of anti-SSB/La autoantibody secreted by individual B cells in both cohorts.

## Discussion

In the present study, we applied SCAN as a single-cell technology to identify and enumerate the frequency of individual B cells secreting anti-SSA/Ro60 and anti-SSB/La autoantibodies in pSS/SLE patients. The results indicated that SCAN technology was capable of detecting isotype-specific anti-SSA/Ro60 and anti-SSB/La autoantibodies from single B cells. In addition, SCAN technology was able to quantify both the frequency and levels of secreting anti-SSA/Ro60 and anti-SSB/La from B cells. Our data indicate that patients exhibited higher frequencies of IgG-specific anti-SSA/Ro60- and anti-SSB/La-producing B cells in comparison to healthy subjects. In addition, patients secreted higher concentrations of anti-SSA/Ro60 autoantibody and similar levels of anti-SSB/La autoantibody compared with the control subjects. These results demonstrate that SCAN technology is highly specific in discriminating different autoantibodies; moreover, it quantitatively determines the level or concentration from a single B cell, which, to our knowledge, was examined for the first time in this study.

Anti-SSA/Ro60 and anti-SSB/La are shown to correlate with a number of clinical symptoms in pSS and SLE [20–24]. Although the data are not definite, patients with pSS with anti-SSA/Ro60 and anti-SSB/La tend to have longer disease duration, parotid gland enlargement, decreased salivary flow, and more severe minor salivary gland infiltration [21]. Additionally, seropositive patients have been reported to manifest a higher prevalence of extraglandular manifestations. Halse et al. [11] found that labial salivary glands (LSG) B cells of patients with pSS produced antibodies against SSA/Ro60 predominantly in the IgG isotype and that these patients also showed high focus scores ≥7 in LSG biopsies. In a subsequent study [25], that research group examined larger cohorts using peripheral blood in which the results indicated that PBMCs of both healthy control subjects and patients with pSS contained significant number of Ig-secreting cells, with similar frequencies of IgG, IgA, and IgM isotypes. However, only 3 of 18 patients were positive for IgG-specific anti-SSA/Ro52, and 1 of 18 patients and 1 of 16 were positive for IgG-anti-SSA/Ro60 and IgG-anti-SSB/La, respectively. Only 1 of 12 patients examined was positive for IgM-anti-SSB/La. Interestingly, the three patients who were positive for IgG-specific anti-SSA/Ro52 showed a correlation with severe disease by having a focus score >8 in LSG biopsies. These two studies suggested that IgG-specific anti-SSA/Ro60 or anti-SSA/Ro52 in LSG or PBMCs correlate with the severity of sialadenitis. Recent studies have supported that these autoantibodies can be used to predict SS up to 20 years before diagnosis [8, 9]. SCAN analysis indicated that individual B cells of patients secreted higher frequencies of anti-SSA/Ro60 and anti-SSA/Ro52, as well as a higher concentration of anti-SSA/Ro60; however, the expression of these autoantibodies did not correlate with disease activity. Additional studies with larger cohorts might be able to resolve this association.

Traditional methods used to measure antibodies in serum have provided critical data for patient diagnosis and prognosis. Other emerging methods, such as proteomic microarray and solution phase luciferase immunoprecipitation systems (LIPS) technology, are used to detect antibody responses to both linear and conformational epitopes, a capability surpassing traditional techniques [26, 27]. The developers of SCAN technology took a different approach by examining the production of autoantibodies by B cells at the single-cell level. SCAN technology is an autoantibody multiplex platform that can massively and simultaneously analyze multiple antibodies from the viable single-cell source. Our data demonstrate that SCAN technology exhibits high sensitivity and specificity by discriminating the presence of anti-SSA/Ro60 and anti-SSB/La autoantibodies. One advantage over ELISA of the technique developed here for estimating the frequency and concentration of an antibody is that the relative occupancy of the antibody is measured using antigen in solution. This approach minimizes confounding multivalent interactions of antibodies with plate-bound antigen. Furthermore, SCAN technology is used to examine the secreted products at the B-cell source, while ELISA measures antibodies present in sera. These fundamental differences potentially limit a direct comparison between ELISA and SCAN technology. The refinement of SCAN technology in terms of practicality and feasibility with comparison with more sensitive assays, such as LIPS or enzyme-linked immunospot, could provide meaningful clinical patient data.

The results of this study provide a proof of concept in that measuring secreting function of individual B cells can provide a more extensive analysis, specifically the frequency, concentration, and isotype of antibody-producing B cells concomitantly, which traditional methods are not capable of. Our results demonstrate that SCAN technology was able to detect anti-SSA/Ro60- and anti-SSB/La-producing B cells in some patients and healthy subjects who were seronegative for anti-SSA/Ro60 and anti-SSB/La. The high frequency and concentration of anti-SSB/La detected by SCAN technology in healthy subjects are interesting and need further investigation. Satoh et al. [10] showed that ANA prevalence in the U.S. population ages 12 years and older was 13.8 % with anti-Ro at 3.9 % and anti-La at a lower percentage. Moreover, healthy control subjects in this study were screened for SLE or pSS, but other potential causes of autoantibody production could be certain infections, cancers, and drugs [28]. Although somewhat unlikely, we cannot rule out the possible cross-reaction between SS-B/La and any unknown environmental antigen. Therefore, it is essential that additional studies with larger cohorts of healthy subjects and pSS/SLE patients are needed to reexamine and validate this finding. More importantly, a larger cohort study will establish a threshold frequency of individual autoantibody-producing B cells to differentiate healthy individuals from those with disease.

## Conclusions

In the present study, we demonstrated that SCAN technology was capable of detecting individual anti-SSA/Ro60- and anti-SSB/La-producing B cells with high specificity. At a single-cell level, the results indicated that patients produced higher levels of autoantibodies at increased frequencies than healthy subjects. SCAN technology has a unique advantage in that it can be used to profile a number of parameters simultaneously which traditional techniques cannot. Currently, SCAN technology has not yet been validated for the classification of patients. Future studies with larger cohort populations will support the findings of the present study and, more importantly, will provide a better understanding on the association between clinical disease and immunological function of autoantibody-producing B cells at the single-cell level.

## Ethics, consent, and permissions

All participants consented to participate in the study. The consent forms are held by the authors and are available for review by the Editor-in-Chief of this journal.

## Abbreviations

AF: Alexa Fluor
ANA: antinuclear autoantibody
BF: bright field
ELISA: enzyme-linked immunosorbent assay
IgG: immunoglobulin G
LIPS: luciferase immunoprecipitation systems
LSG: labial salivary glands
MFI: mean fluorescence intensity
PBMC: peripheral blood mononuclear cell
PDMS: polydimethylsiloxane
pSS: primary Sjögren’s syndrome
RF: rheumatoid factor
SCAN: single-cell antibody nanowell
SICCA: Sjögren’s International Collaborative Clinical Alliance
SLE: systemic lupus erythematosus
SS: Sjögren’s syndrome
sSS: secondary Sjögren’s syndrome

## References

[1] Jonsson R, Haga HJ, Gordon TP (2000). Current concepts on diagnosis, autoantibodies and therapy in Sjögren’s syndrome. Scand J Rheumatol.

[2] Cornec D, Jamin C, Pers JO (2014). Sjögren’s syndrome: where do we stand, and where shall we go?. J Autoimmun..

[3] Dawson L, Tobin A, Smith P, Gordon T (2005). Antimuscarinic antibodies in Sjögren’s syndrome: where are we, and where are we going?. Arthritis Rheum.

[4] Dawson LJ, Fox PC, Smith PM (2006). Sjögrens syndrome—the non-apoptotic model of glandular hypofunction. Rheumatology (Oxford).

[5] Sawalha AH, Harley JB (2004). Antinuclear autoantibodies in systemic lupus erythematosus. Curr Opin Rheumatol.

[6] Harley JB, Alexander EL, Bias WB, Fox OF, Provost TT, Reichlin M (1986). Anti-Ro (SS-A) and anti-La (SS-B) in patients with Sjögren’s syndrome. Arthritis Rheum.

[7] Routsias JG, Tzioufas AG (2007). Sjögren’s syndrome—study of autoantigens and autoantibodies. Clin Rev Allergy Immunol.

[8] Jonsson R, Theander E, Sjöström B, Brokstad K, Henriksson G (2013). Autoantibodies present before symptom onset in primary Sjögren syndrome. JAMA.

[9] Theander E, Jonsson R, Sjöström B, Brokstad K, Olsson P, Henriksson G (2015). Prediction of Sjögren’s syndrome years before diagnosis and identification of patients with early onset and severe disease course by autoantibody profiling. Arthritis Rheumatol.

[10] Satoh M, Chan EK, Ho LA, Rose KM, Parks CG, Cohn RD (2012). Prevalence and sociodemographic correlates of antinuclear antibodies in the United States. Arthritis Rheum.

[11] Halse A, Harley JB, Kroneld U, Jonsson R (1999). Ro/SS-A-reactive B lymphocytes in salivary glands and peripheral blood of patients with Sjögren’s syndrome. Clin Exp Immunol.

[12] Maier-Moore JS, Koelsch KA, Smith K, Lessard CJ, Radfar L, Lewis D (2014). Antibody-secreting cell specificity in labial salivary glands reflects the clinical presentation and serology in patients with Sjögren’s syndrome. Arthritis Rheumatol.

[13] Egner W (2000). The use of laboratory tests in the diagnosis of SLE. J Clin Pathol.

[14] Hanly JG, Su L, Farewell V, Fritzler MJ (2010). Comparison between multiplex assays for autoantibody detection in systemic lupus erythematosus. J Immunol Methods.

[15] Mahler M, Meroni PL, Bossuyt X, Fritzler MJ (2014). Current concepts and future directions for the assessment of autoantibodies to cellular antigens referred to as anti-nuclear antibodies. J Immunol Res..

[16] Satoh M, Tanaka S, Chan EK (2015). The uses and misuses of multiplex autoantibody assays in systemic autoimmune rheumatic diseases. Front Immunol..

[17] Nguyen CQ, Ogunniyi AO, Karabiyik A, Love JC (2013). Single-cell analysis reveals isotype-specific autoreactive B cell repertoires in Sjögren’s syndrome. PLoS One.

[18] Ogunniyi AO, Story CM, Papa E, Guillen E, Love JC (2009). Screening individual hybridomas by microengraving to discover monoclonal antibodies. Nat Protoc.

[19] Story CM, Papa E, Hu CC, Ronan JL, Herlihy K, Ploegh HL (2008). Profiling antibody responses by multiparametric analysis of primary B cells. Proc Natl Acad Sci U S A.

[20] Billaut-Mulot O, Cocude C, Kolesnitchenko V, Truong MJ, Chan EK, Hachula E (2001). SS-56, A novel cellular target of autoantibody responses in Sjögren syndrome and systemic lupus erythematosus. J Clin Invest.

[21] Bournia VK, Vlachoyiannopoulos PG (2012). Subgroups of Sjögren syndrome patients according to serological profiles. J Autoimmun.

[22] Gordon TP, Bolstad AI, Rischmueller M, Jonsson R, Waterman SA (2001). Autoantibodies in primary Sjögren’s syndrome: new insights into mechanisms of autoantibody diversification and disease pathogenesis. Autoimmunity.

[23] Tzioufas AG, Wassmuth R, Dafni UG, Guialis A, Haga HJ, Isenberg DA (2002). Clinical, immunological, and immunogenetic aspects of autoantibody production against Ro/SSA, La/SSB and their linear epitopes in primary Sjögren’s syndrome (pSS): a European multicentre study. Ann Rheum Dis.

[24] Damoiseaux J, Andrade LE, Fritzler MJ, Shoenfeld Y (2015). Autoantibodies 2015: from diagnostic biomarkers toward prediction, prognosis and prevention. Autoimmun Rev.

[25] Halse A, Wahren-Herlenius M, Jonsson R (1999). Ro/SS-A- and La/SS-B-reactive B lymphocytes in peripheral blood of patients with Sjögren’s syndrome. Clin Exp Immunol.

[26] Hu S, Vissink A, Arellano M, Roozendaal C, Zhou H, Kallenberg CG (2011). Identification of autoantibody biomarkers for primary Sjögren’s syndrome using protein microarrays. Proteomics.

[27] Volchenkov R, Jonsson R, Appel S (2012). Anti-Ro and anti-La autoantibody profiling in Norwegian patients with primary Sjögren’s syndrome using luciferase immunoprecipitation systems (LIPS). Scand J Rheumatol.

[28] Satoh M, Chan EK, Sobel ES, Kimpel DL, Yamasaki Y, Narain S (2007). Clinical implication of autoantibodies in patients with systemic rheumatic diseases. Expert Rev Clin Immunol.

